# The Ubiquitin/Proteasome System Mediates Entry and Endosomal Trafficking of Kaposi's Sarcoma-Associated Herpesvirus in Endothelial Cells

**DOI:** 10.1371/journal.ppat.1002703

**Published:** 2012-05-17

**Authors:** Whitney Greene, Wei Zhang, Meilan He, Colleen Witt, Fengchun Ye, Shou-Jiang Gao

**Affiliations:** 1 Tumor Virology Program, Greehey Children's Cancer Research Institute, and Department of Pediatrics, University of Texas Health Science Center San Antonio, San Antonio, Texas, United States of America; 2 Department of Molecular Microbiology and Immunology, Keck School of Medicine, University of Southern California, Los Angeles, California, United States of America; 3 Department of Biology, College of Sciences, University of Texas at San Antonio, San Antonio, Texas, United States of America; University of Texas-Southwesten Medical school, United States of America

## Abstract

Ubiquitination, a post-translational modification, mediates diverse cellular functions including endocytic transport of molecules. Kaposi's sarcoma-associated herpesvirus (KSHV), an enveloped herpesvirus, enters endothelial cells primarily through clathrin-mediated endocytosis. Whether ubiquitination and proteasome activity regulates KSHV entry and endocytosis remains unknown. We showed that inhibition of proteasome activity reduced KSHV entry into endothelial cells and intracellular trafficking to nuclei, thus preventing KSHV infection of the cells. Three-dimensional (3-D) analyses revealed accumulation of KSHV particles in a cytoplasmic compartment identified as EEA1+ endosomal vesicles upon proteasome inhibition. KSHV particles are colocalized with ubiquitin-binding proteins epsin and eps15. Furthermore, ubiquitination mediates internalization of both KSHV and one of its receptors integrin β1. KSHV particles are colocalized with activated forms of the E3 ligase c-Cbl. Knock-down of c-Cbl or inhibition of its phosphorylation reduced viral entry and intracellular trafficking, resulting in decreased KSHV infectivity. These results demonstrate that ubiquitination mediates internalization of both KSHV and one of its cognate receptors integrin β1, and identify c-Cbl as a potential E3 ligase that facilitates this process.

## Author Summary

Ubiquitination, a post-translational modification, mediates important cellular functions including endocytic transport of molecules. Kaposi's sarcoma-associated herpesvirus (KSHV) is a gammaherpesvirus linked to the development of Kaposi's sarcoma, an endothelial malignancy commonly found in AIDS patients, and several other malignancies. KSHV enters endothelial cells primarily through clathrin-mediated endocytosis. In this study, we show that the proteasome activity is required for KSHV entry into endothelial cells and intracellular trafficking to nuclei. Inhibition of proteasome activity reduced KSHV infectivity and led to the accumulation of KSHV particles in EEA1+ early endosomal vesicles. Furthermore, we show that ubiquitination mediates the internalization of both KSHV and one of its receptors integrin β1. KSHV particles are colocalized with ubiquitin-binding proteins epsin and eps15, as well as activated forms of the E3 ligase c-Cbl. Knock-down of c-Cbl or inhibition of its phosphorylation blocked KSHV entry and trafficking, thus preventing KSHV infection of endothelial cells. Together, these results illustrate the essential role of ubiquitination during the internalization of KSHV and its cognate receptor integrin β1. The identification of an E3 ligase that mediates the ubiquitination of KSHV and its cognate receptor integrin β1 leading to viral entry provide a potential therapeutic target for this oncogenic virus.

## Introduction

Ubiquitination has been linked to diverse cellular functions including directing protein recycling [1], [2]. Addition of four or more ubiquitin moieties (polyubiquitination) provides the necessary signal for targeting protein for proteasomal degradation. Ubiquitination of target cell surface membrane proteins triggers its internalization and endocytic sorting in addition to proteasomal degradation. For examples, monoubiquitination, the addition of single ubiquitin moieties, of epidermal growth factor receptor (EGFR) cytoplasmic tail induces its internalization and transport to the lysosome for degradation [3]–[5] while ubiquitination of membrane receptors β2 adrenergic receptor and interleukin 2 receptor β chain is required for their correct internalization and processing [6]–[8].

The two arms of the ubiquitin/proteasome system are separate, yet closely linked [9]. Ubiquitination is initiated by the conjugation of two ubiquitin molecules to the E1 activating enzyme. The ubiquitin molecules are then transferred to the E2 conjugating enzyme in an ATP-dependent reaction. E2 enzyme interacts with a specific E3 partner and transfers the ubiquitin molecules to the target protein [9]. The ubiquitinated proteins are subsequently transported to the proteasome where the ubiquitin chains are cleaved off by deubiquitinating enzymes (DUBs) releasing the ubiquitin molecules to reenter the cycle [2]. Mammalian cells express two E1 activating enzymes, up to 40 E2 conjugating enzymes, and hundreds of E3 ligases [10], [11].

Kaposi's sarcoma-associated herpesvirus (KSHV) primarily utilizes the clathrin-mediated endocytosis pathway to enter host cells [12], [13]. KSHV is a large enveloped DNA virus that uses cell surface molecules as its receptors, which includes heparan sulfate [14], integrin α3β1 [15], integrin α×β3 [16], xCT [17] and DC-SIGN [18], to initiate entry into target cells. The possible role of the ubiquitin/proteasome system during the internalization of KSHV and its cognate receptor(s) has yet to be examined. In this study, we investigated the role of the ubiquitin/proteasome system during the entry and intracellular trafficking of KSHV and one of its cognate receptors, integrin β1. We detailed the requirements for proteasome function and de novo ubiquitination for efficient viral entry into endothelial cells and intracellular trafficking to the perinuclear regions. Inhibition of proteasome function by several chemicals reduced viral entry and intracellular trafficking, and caused an accumulation of viral particles in an early endosomal compartment. As a result, KSHV infectivity was reduced. Inhibition of proteasome function resulted in an enrichment of ubiquitinated proteins and a depletion of the cellular pool of free ubiquitin. Inhibition of E1 activating enzyme reduced internalization of KSHV as well as its receptor integrin β1. The E3 ligase c-Cbl was activated during KSHV entry. Knock-down of c-Cbl or inhibition of its activation blocked KSHV entry and intracellular trafficking, resulting in reduced viral infectivity, suggesting that this E3 ligase may mediate the internalization, and endosomal transport and sorting of KSHV-containing endosomes. We also report the involvement of the E3 ligase Rabex5, and the endocytic adaptor proteins epsin and eps15, during KSHV entry into endothelial cells. These results suggest that ubiquitination is required for KSHV entry into endothelial cells and during its endosomal sorting and intracellular trafficking.

## Results

### Inhibition of Proteasome Function Reduces KSHV Entry into Endothelial Cells and Intracellular Trafficking to Nuclei

**Figure 1:**
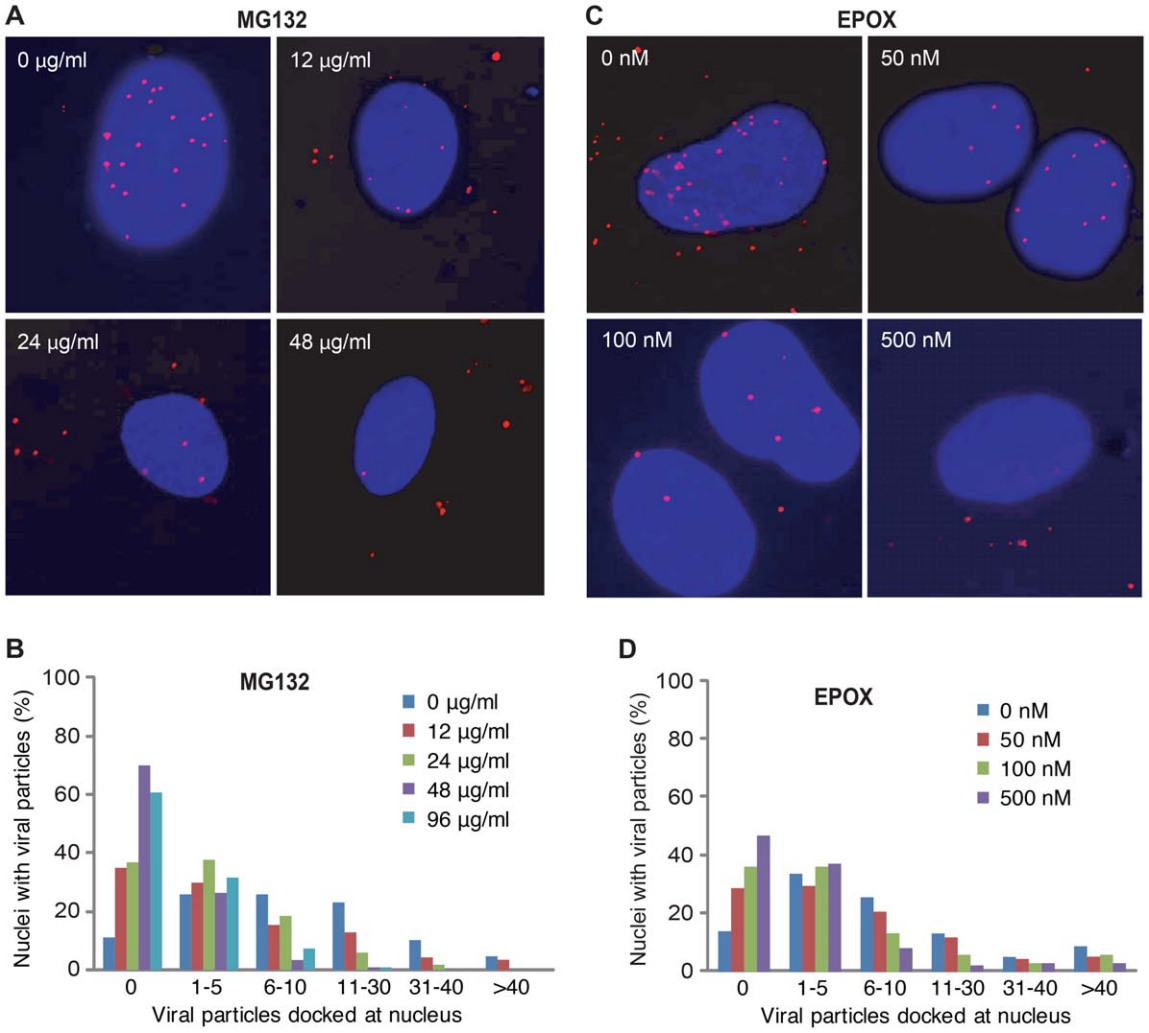
Inhibition of proteasome activity prevents KSHV entry into endothelial cells and intracellular trafficking to nuclei. (A and C) HUVEC were treated with proteasome inhibitors MG132 (A) and EPOX (C) at indicated doses for 1 hr. Cells were then infected with KSHV in the presence of the inhibitors, fixed at 4 hpi, and stained for Orf65+ viral particles (red) and nuclei (blue). (B and D) Quantification of the total number of Orf65+ particles docked at each nucleus following treatment with proteasome inhibitors MG132 (B) and EPOX (D).

**Figure 2:**
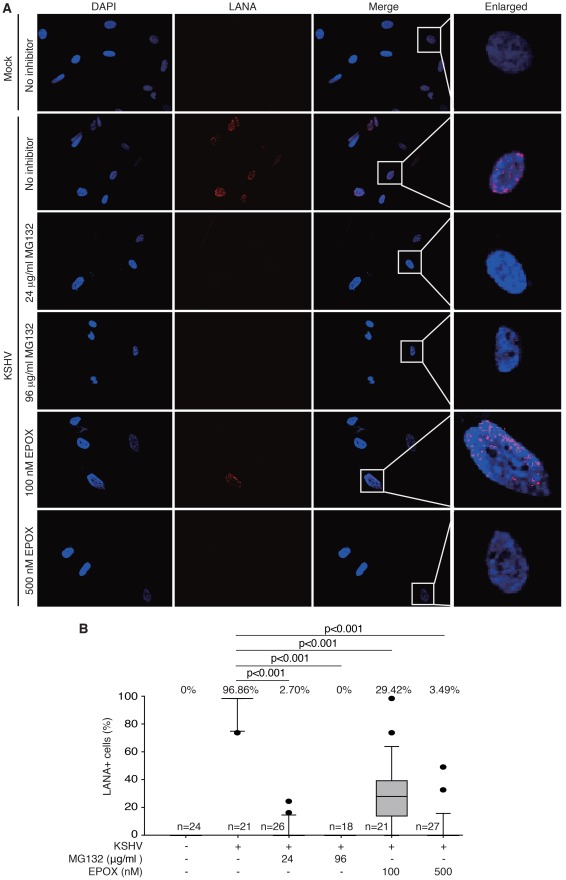
Inhibition of proteasome activity reduced KSHV infectivity in endothelial cells. (A) Proteasome inhibitors MG132 and EPOX reduced the numbers of LANA-positive cells. HUVEC grown on coverslips were pretreated with proteasome inhibitors for 1 hr and infected with KSHV for 4 hr in the presence of the inhibitors. Cells were fixed and stained for LANA (red) and nuclei (blue) at 48 hpi. (B) Analyses of LANA-positive cells following treatment with proteasome inhibitors depicted as box and whisker plots. Open boxes indicate the 75th and 25th percentiles. Top and bottom short lines indicate the 90th and 10th percentiles, respectively. Middle lines in the boxes indicate the medians, also shown in “%” on tops. Outliers outside the 90th and 10th percentiles are represented as black dots. Some elements of the box and whisker plots are missing because of overrepresentation at lower or higher percentiles. “n” represents total number of fields analyzed per sample. *p*-values <0.05 are statistically significant.

To investigate the role of proteasome activity during KSHV entry and intracellular trafficking, primary human umbilical vein endothelial cells (HUVEC) were pretreated with chemical inhibitors of the 26S proteasome, MG132 or epoxomicin (EPOX), for 1 hr prior to KSHV infection. MG132 is a peptide aldehyde that potently inhibits the transition state of the proteasome [19]. EPOX selectively and irreversibly binds to the catalytic β subunits of the proteasome and effectively inhibits multiple proteolytic activities [20]. At 4 hr post-infection (hpi), the total numbers of viral particles docked at each nucleus or in a whole cell revealed by staining for small capsid protein Orf65 were quantified. Inhibition of proteasome activity with either MG132 or EPOX had no effect on the total numbers of cell-associated viral particles including those that were associated with plasma membrane (Figure S1), indicating that the proteasome inhibitors do not affect the attachment of viral particles to the cells. However, inhibition of proteasome activity with either MG132 (Figure 1A–B) or EPOX (Figure 1C–D) resulted in significantly fewer Orf65+ viral particles docked at the nuclear membranes at a dose-dependent fashion compared to cells treated with DMSO alone. Under these experimental conditions, we did not observe any noticeable cytotoxicity of the inhibitors to the cells based on propidium iodide (PI) staining (data not shown). To determine whether proteasome inhibitors actually blocked the infectious pathway of KSHV entry and intracellular trafficking, we stained the cells for the expression of LANA protein (Orf73) for evidence of successful infection. Inhibition of proteasome function significantly reduced the numbers of LANA-positive cells at 48 hpi (Figure 2A and B). At the highest used doses of MG132 (96 µg/ml) and EPOX (500 nM), LANA-positive cells were reduced from 96.86% of the untreated cells to 0% and 3.49%, respectively (Figure 2B). These results indicate that proteasome activity is necessary for successful KSHV intracellular trafficking to the nuclei.

**Figure 3:**
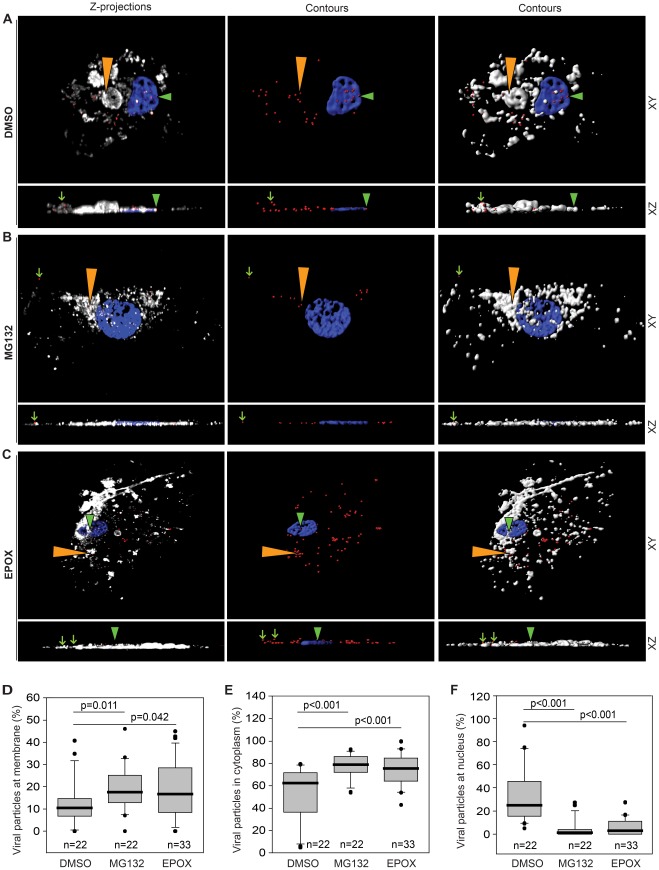
Cellular localization of KSHV particles following inhibition of proteasome activity. (A, B and C) HUVEC treated with DMSO (A), MG132 (B) or EPOX (C) were incubated with KSHV for 4 hr, stained for viral particles (red), cell membrane (white) and nuclei (blue). Z-stack images were acquired with confocal laser-scanning microscopy. Three-dimensional software was used to generate z-projection images (left column). Three-dimensional contoured images (middle and right columns) were generated using Imaris image analysis software to determine the localizations of KSHV particles in relation to the cell membrane, the cell interior, and the cell nucleus (upper panels). The 3-D images in the upper panels were rotated on the x-axis to visualize viral particles attached at the cell membrane (small green arrows), internalized into the cytoplasmic space (orange triangles), or docked at the nucleus (green triangles) (lower panels). Videos S1, S2, and S3 correspond to Figures 3A, B, and C respectively. Video S4 depicts viral particles that have been engulfed, and are enclosed within a membrane-bound vesicle. (D–F) Quantification of cellular localization of cell-associated KSHV particles as illustrated in A, B, and C, respectively. Box and whisker plots depict the statistical analyses of the cellular localization of KSHV particles. Open boxes indicate the 75th and 25th percentiles. Top and bottom short lines indicate the 90th and 10th percentiles, respectively. Middle thick lines in the boxes indicate the medians. Outliers outside the 90th and 10th percentiles are represented as black dots. To ensure representation, only cells with at least five particles per cell were counted. The percentages of KSHV particles retained at the membrane for each sample are illustrated in (D), the percentages of internalized KSHV particles in the cytoplasmic space are depicted in (E), and the percentages of KSHV particles docked at the nuclei are illustrated in (F). “n” represents total number of cells analyzed per sample. *p*-values <0.05 are statistically significant.

We next sought to identify where viral particles were trapped in cells treated with proteasome inhibitors. HUVEC were pretreated with the indicated inhibitors for 1 hr and then inoculated with KSHV in the presence of the inhibitors. At 4 hpi, cells were stained for viral capsids (Orf65) in red, the cell membrane (AlexaFluor 647 WGA) in white, and nuclei (DAPI) in blue. Confocal laser-scanning microscopy was used to acquire Z-stack images. Deconvolved images were analyzed in three-dimensional (3-D) to determine the numbers of viral particles in the extracellular, membrane-bound, cytoplasmic, or nuclear spaces. The spot detection function was used to identify and quantify the number of Orf65+ viral particles in a cell. Surface contours were generated to enable the visualization of cell membranes and nuclei (Figures 3A–C, and Videos S1–S4). There is a modest but statistically significant increase in the numbers of viral particles retained at the plasma membranes following proteasome inhibition (Figure 3D). In addition, a greater proportion of viral particles were retained in the cytoplasm in the proteasome-inhibited cells compared to untreated cells (Figure 3E). As expected, fewer viral particles reached the nuclear membrane (Figure 3F).

**Figure 4:**
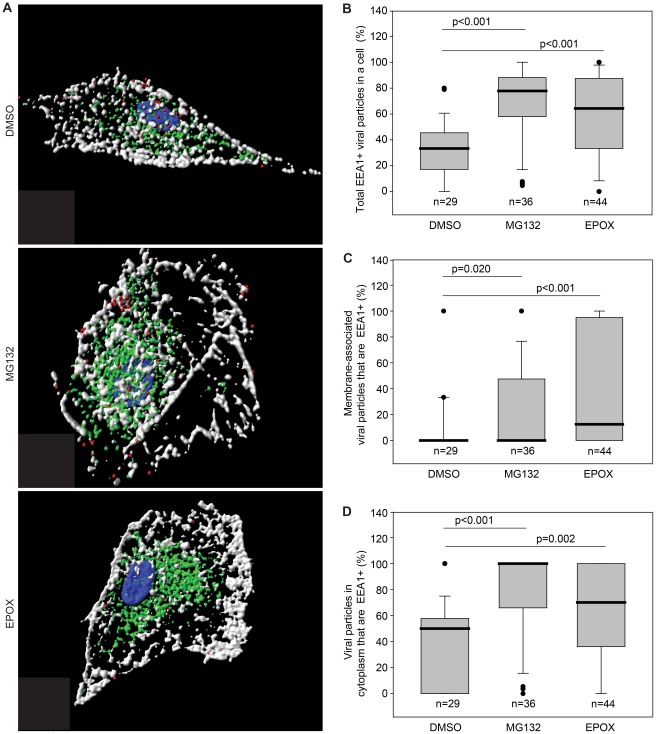
Proteasome inhibitors increase KSHV particles associated with early endosomal marker EEA1. (A) HUVEC treated with DMSO, MG132 or EPOX were incubated with KSHV for 4 hr, stained for viral particles (red), EEA1 (green), membrane (white), and nuclei (blue). Z-stacks were deconvolved, and Imaris image analysis software was used to generate 3-D contoured images. Videos S5, S6 and S7 correspond to cells treated with DMSO, MG132 or EPOX, respectively, labeled for KSHV particles and EEA1 rotated around the x-axis. Video S8 depicts KSHV particles enclosed in an EEA1+ vesicle. (B–D) Imaris 3-D colocalization analyses determine the total numbers of KSHV particles in a cell that are colocalized with EEA1 (B), the numbers of viral particles localized at cell membranes that are EEA1+ (C), and the numbers of viral particles localized within the cytoplasmic spaces that are EEA1+ (D). Box and whisker plots depict the statistical analyses of the cellular localization of KSHV particles as described in Figure 3.

**Figure 5:**
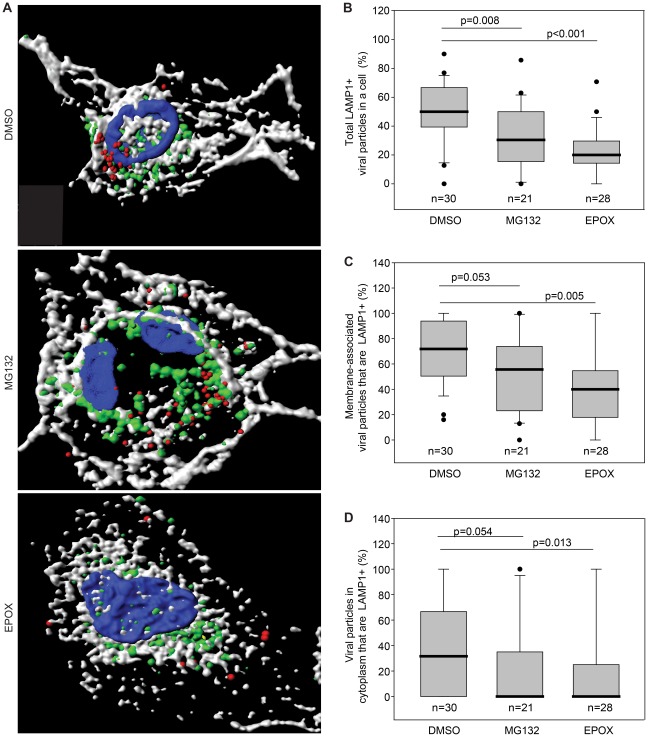
Inhibition of proteasome function reduces KSHV particles associated with late endosomal/lysosomal marker LAMP1. (A) HUVEC treated with DMSO, MG132 or EPOX were incubated with KSHV for 4 hr, stained for KSHV particles (red), LAMP1 (green), membrane (white), and nuclei (blue). Z-stacks were deconvolved and, Imaris image analysis software was used to generate 3-D contoured images. Videos S9, S10 and S11 correspond to cells treated with DMSO, MG132 or EPOX, respectively, labeled for KSHV particles and LAMP1 rotated around the x-axis. Video S12 depicts KSHV particles enclosed in an LAMP1+ vesicle. (B–D) Imaris 3-D colocalization analyses determine the total numbers of KSHV particles in a cell that are colocalized with LAMP1 (B), the numbers of viral particles localized at cell membranes that are LAMP1+ (C), and the numbers of viral particles localized within the cytoplasmic spaces that are LAMP1+ (D). Box and whisker plots depict the statistical analyses of the cellular localization of KSHV particles as described in Figure 3.

The increased number of viral particles in the cytoplasm suggests that inhibition of proteasome activity might lead to an arrest in the maturation of viral particle-containing endosomes. To identify the specific cellular compartment where the viral particles were arrested, HUVEC were inoculated with KSHV and fixed at 4 hpi. In addition to Orf65+ viral particles, cells were stained for either EEA1 (Figure 4, and Videos S5–S8) or LAMP1 (Figure 5, and Videos S9–S12) to identify the early endosomal compartment or the late endosome/lysosomal compartment, respectively. Confocal z-stack images were acquired and subjected to 3-D colocalization analysis. Proteasome inhibition resulted in more viral particles colocalized with the early endosome marker, EEA1, in the entire cells (Figure 4B), or viral particles associated with plasma membrane (Figure 4C) and cytoplasm (Figure 4D), compared to untreated controls. In contrast, fewer viral particles were colocalized with LAMP1, the late endosome/lysosome marker, in the entire cells (Figure 5B), or viral particles associated with plasma membrane (Figure 5C) and cytoplasm (Figure 5D), following proteasome inhibition. These results suggest that proteasome function is required for the maturation of virus-containing endosomes from the early to late endosome, and that endosomal maturation is necessary for successful viral trafficking to nuclei.

### KSHV Particles Are Colocalized with Ubiquitin-Binding Proteins during Internalization

**Figure 6:**
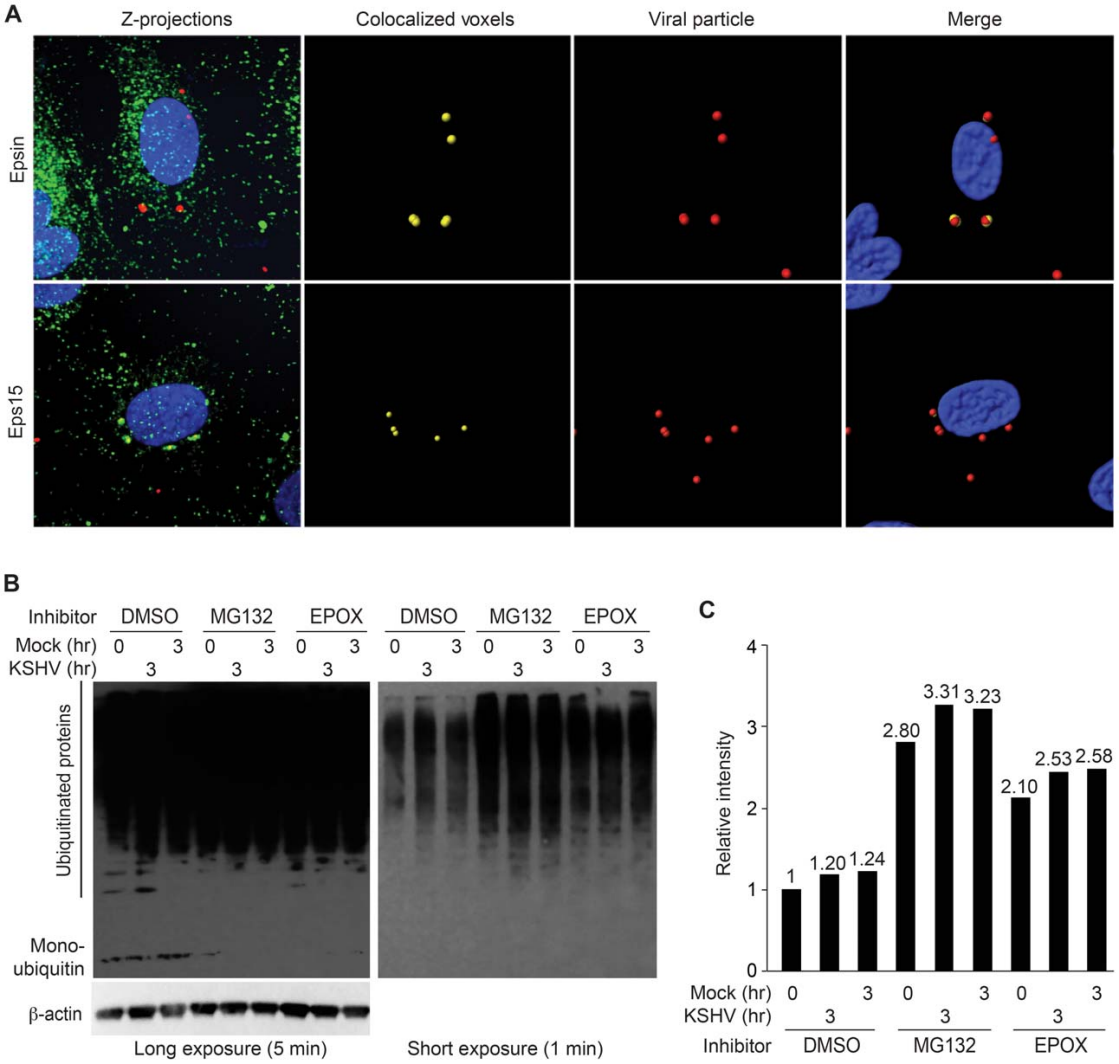
Association of KSHV particles with ubiquitin-interacting proteins during entry into endothelial cells. (A) Colocalization of KSHV particles with Epsin and Eps15 during entry into HUVEC. HUVEC infected with KSHV for 1 hr were stained for viral particles (red) and ubiquitin-interacting proteins (green) Epsin (top) or Eps15 (bottom). Images were analyzed in 3-D for colocalization of KSHV particles with Epsin or Eps15. Regions of colocalization are depicted in yellow (second column). (B) Inhibition of proteasome function depletes the cellular pool of free ubiquitin. HUVEC were treated with DMSO, MG132, or EPOX for 1 hr, and then infected with KSHV for 3 hr. Cell lysates were collected and analyzed by immunoblotting to detect both low molecular weight free ubiquitin as well as ubiquitinated proteins (top panel). β-actin was used as a loading control (bottom panel). Longer exposure shown on the left was used to detect the low molecular weight band corresponding to monomeric ubiquitin at 7.5 kD. The shorter exposure shown at the right panel was used to illustrate relative levels of ubiquitinated proteins. (C) Quantification of relative levels of ubiquitinated proteins detected in B.

Proteasome function *per se* may not directly regulate internalization of KSHV particles. Inhibition of proteasome function results in the accumulation of ubiquitinated proteins, which may reduce the pool of free ubiquitin available for *de novo* ubiquitination events, implying that ubiquitination of KSHV and/or its receptors could be the mediator of KSHV internalization. To further understand how proteasome inhibition prevents viral entry and intracellular trafficking, we investigated the role of two adaptor molecules that link clathrin-mediated endocytosis and ubiquitination. Epsin and Eps15 are endocytic adaptor proteins that recognize ubiquitinated cargo through their ubiquitin-interacting motifs (UIMs) [21], [22]. Binding of Epsin to ubiquitinated EGFR is required for its translocation into clathrin-coated pits [22]. Binding of Eps15 to ubiquitinated Cx43 mediates its internalization through endocytosis [21]. Since KSHV enters HUVEC primarily through clathrin-mediated endocytosis, we hypothesized that endocytic adaptor proteins such as Epsin or Eps15 may play an important role during KSHV entry. As shown in Figure 6A, most KSHV particles were colocalized with both Epsin and Eps15 during infection of HUVEC. However, we also observed small numbers of KSHV particles that were not colocalized with the adaptor proteins, possibly reflecting the transient nature of the interaction. The detection of these non-colocalized particles indicates that the observed colocalization is specific but not due to random distribution of the viral particles. These results suggest that KSHV utilizes clathrin-mediated endocytosis to enter cells, and either KSHV or its receptor(s) is subjected to ubiquitination during the viral internalization process.

### Inhibition of Proteasome Function Depletes the Cellular Pool of Free Ubiquitin

Although the proteasome primarily functions as a protein degradation system, inhibition of its functions has deleterious effects on many other cellular activities, including endocytosis [23], [24]. We hypothesized that inhibition of proteasome function results in an accumulation of ubiquitinated proteins and thus a reduction in the cellular pool of ubiquitin monomers available for *de novo* ubiquitination reactions. To test this, we treated endothelial cells with either MG132 or EPOX for 1 hr, before the inoculation with KSHV. Cells were analyzed for ubiquitin by immunoblots at different times post-treatment. Both polyubiquitinated proteins and free ubiquitin were detected in the DMSO-treated samples regardless of KSHV infection (Figure 6B–C). The typical smear of higher molecular weight ubiquitinated proteins was visible in all lysates tested, though there was an obvious increase in the amount of ubiquitination in the samples treated with proteasome inhibitors. However, low molecular weight free ubiquitin was not detected in samples treated with proteasome inhibitors, indicating that inhibition of proteasome function reduces the availability of ubiquitin monomers.

### Ubiquitination Is Required for Internalization of KSHV Receptor Integrin β1

**Figure 7:**
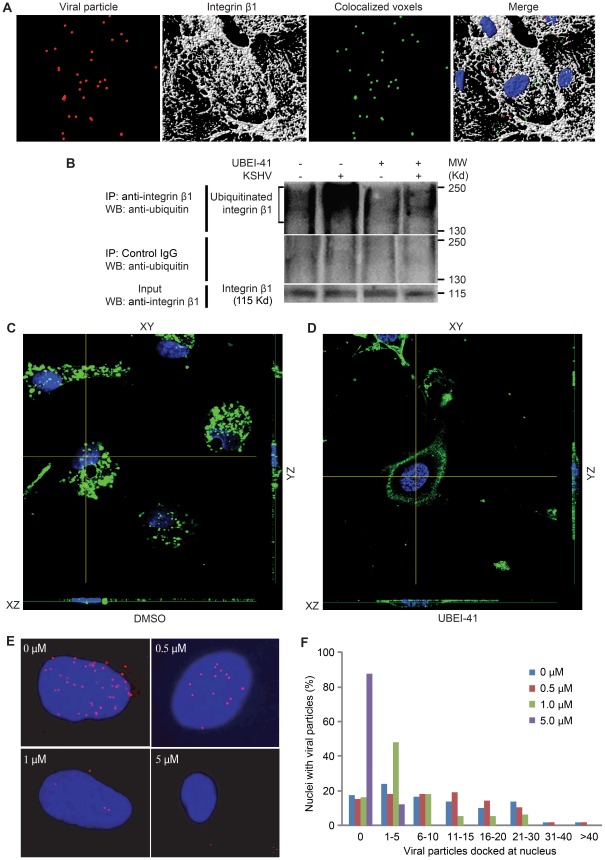
Ubiquitination mediates internalization of integrin β1 and KSHV entry and intracellular trafficking. (A) Colocalization of KSHV particles with integrin β1 during entry into endothelial cells. HUVEC infected with KSHV were stained for viral particles (red) and integrin β1 (white). Images were subjected to 3-D colocalization analysis. Regions of colocalization are depicted in green. (B) Increase of ubiquitination of integrin β1 following KSHV infection, which is blocked by UBEI-41. HUVEC pretreated with DMSO or UBEI-41 for 1 hr were infected with KSHV. At 1 hpi, integrin β1 was immunoprecipitated and the ubiquitinated protein detected by Western-blotting. (C and D) Ubiquitination mediates internalization of integrin β1. HUVEC pretreated with DMSO (C) or UBEI-41 (D) for 1 hr were labeled with a monoclonal antibody to integrin β1 for 1 hr, fixed and labeled with an AlexaFluor488 goat anti-mouse antibody. Z-stack images were acquired to visualize the cellular location of the labeled integrin β1. (E) Inhibition of E1 activating enzyme reduces KSHV entry and intracellular trafficking in endothelial cells. HUVEC were pretreated for 1 hr with UBEI-41 before infection with KSHV for 4 hr, and stained for viral particles (red) and nuclei (blue). (F) Quantification of the numbers of KSHV particles docked at nuclei following inhibition with UBEI-41 as described in E.

Integrin α3β1 is one of the cellular receptors for KSHV entry into cells [15]. As expected, 3-D colocalization analysis revealed significant levels of colocalization between KSHV particles with integrin β1 (Figure 7A). To determine the specificity of the observed colocalization, we examined the colocalization of integrin β1 with transferrin, cholera toxin B and rhesus rhadinovirus, all of which enter cells by endocytosis without using integrin β1 as a receptor [25], [26]. In contrast to the perfect colocalization of KSHV, less than 10% of RRV, transferrin or cholera toxin B was colocalized with integrin β1 (Figure S2).

Integrins are transmembrane glycoproteins composed of α and β chains. Integrins are constitutively endocytosed and recycled [27], [28]. While integrins are ubiquitinated [29], the role of ubiquitination in integrin endocytosis is not well understood. As a known receptor for KSHV entry into cells, we hypothesized that ubiqutination regulates the internalization of integrin β1 and KSHV. UBEI-41 is an inhibitor of the E1 activating enzyme that potently and specifically blocks the first step of the ubiquitination reaction [30]. Uninfected cells had low level of ubiquitinated integrin β1, which was significantly increased following KSHV infection (Figure 7B). Treatment with UBEI-41 almost completely abolished the level of ubiquitinated integrin β1 in both uninfected and KSHV-infected cells.

To further understand the role of ubiquitination during the internalization of integrin β1, we labeled live cells with a monoclonal antibody against integrin β1 to track its internalization from the plasma membrane. As shown in Figure 7C, integrin β1 was internalized and localized to the perinuclear region in untreated cells. Following treatment with UBEI-41, it was retained at the plasma membrane (Figure 7D), suggesting integrin β1 internalization requires ubiquitination.

### Inhibition of E1 Activating Enzyme Inhibits KSHV Entry and Intracellular Trafficking in Endothelial Cells

The results from Figure 6B suggest that depletion of free ubiquitin upon inhibition of proteasome function may be the mechanism that inhibits the entry and intracellular trafficking of KSHV particles. Since inhibition of E1 activating enzyme activity prevents integrin β1 internalization, we expect KSHV entry and intracellular trafficking to be also affected. Indeed, inhibition of E1 activating enzyme and ubiquitination reduced the number of viral particles that successfully entered the cells and trafficked to the nuclei (Figure 7E–F). However, UBEI-41 had no detectable effect on the total numbers of viral particles per cell including those that were associated with plasma membrane (Figure S1). Thus, the inhibitor did not affect the attachment of viral particles to the cells. PI staining did not detect any cytotoxicity to the cells at all the concentrations used (data not shown). Together, these results indicate that ubiquitination is required for efficient viral entry and intracellular trafficking.

### The Role of E3 Ligases c-Cbl and Rabex5 in KSHV Entry and Intracellular Trafficking

**Figure 8:**
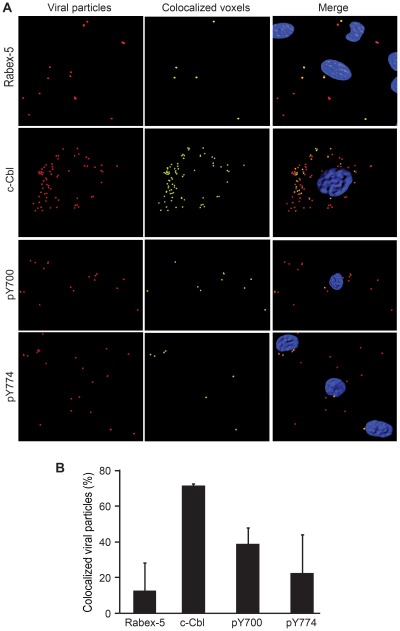
Colocalization of KSHV particles with E3 ligases during KSHV entry and intracellular trafficking in endothelial cells. (A) Most KSHV particles are colocalized with E3 ligase c-Cbl or its activated phosphorylated forms but, to a less extent, with Rabex5. HUVEC infected with KSHV for 4 hr were stained for KSHV particles (red), E3 ligase Rabex5, c-Cbl or its phosphorylated forms (pY700 or pY774) (green), and cell nuclei (blue). Z-stack images were acquired and used for colocalization analysis with the Imaris software. Regions of 3-D colocalization are depicted in yellow. (B) Quantification of colocalization of KSHV particles with Rabex5, c-Cbl, phospho-Y700 c-Cbl and phospho-Y774 c-Cbl.

E3 ubiquitin ligases are the final effectors in the ubiquitination cascade. E3 ligases contain the substrate recognition motifs that provide the target specificity for ubiquitination. Approximately 1,000 E3 ligases have been identified in humans. Among them, Rabex5 is an E3 ligase involved in endocytosis and endosomal maturation by specifically targeting Rab5-positive early endosomes [31], [32] while the E3 ligase c-Cbl ubiquitinates integrin complexes [33]. c-Cbl is activated through phosphorylation by the Src family kinases principally at tyrosine residues 700, 731, and 774 [34]–[36]. Thus, Rabex-5 and c-Cbl are candidate E3 ligases that may potentially mediate KSHV internalization. We therefore sought to investigate the roles Rabex-5 and c-Cbl in KSHV entry into endothelial cells. As shown in Figure 8A–B and Figure S3, only a minimal amount of KSHV particles (6%) were colocalized with Rabex5 during KSHV infection. In contrast, a significant fraction of KSHV particles (70%) were colocalized with c-Cbl. Furthermore, KSHV particles were also colocalized with two activated phosphorylated forms of c-Cbl, at 40% with phospho-tyrosine 700, and at 25% with phospho-tyrosine 774.

**Figure 9:**
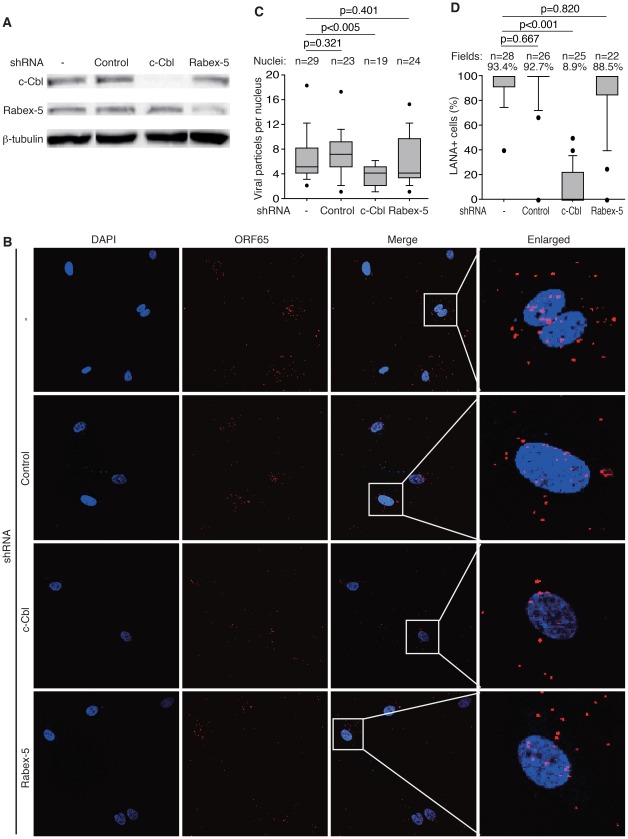
Knock-down of c-Cbl but not Rabex-5 inhibits KSHV entry and intracellular trafficking, and prevents KSHV infection of endothelial cells. (A) HUVEC mock-treated or infected with c-Cbl, Rabex-5 or control shRNA lentivirus particles for 4 days were examined for the expression of c-Cbl, Rabex-5 and β-tubulin by Western-blotting. (B) HUVEC grown on coverslips were mock treated or infected with c-Cbl, Rabex-5 or control lentivirus particles for 4 days, and infected by KSHV for 4 hr. Cells were stained for Orf65+ viral particles (red) and nuclei (blue). Knock-down with c-Cbl shRNA but not Rabex-5 shRNA or control shRNA decreased the numbers of KSHV particles successfully docked at perinuclear regions. (C) Analyses of numbers of KSHV particles docked on each nucleus following shRNA lentivirus infection depicted as box and whisker plots as described in Figure 3. (D) Quantification of LANA-positive cells following KSHV infection for 48 hr in cells with prior knock-down with c-Cbl, Rabex-5, or control shRNA (Figure S4). HUVEC grown on coverslips were mock treated or infected with lentivirus particles for 4 days, and infected by KSHV. At 48 hpi, cells were stained for LANA (red) and nuclei (blue). LANA-positive cells were analyzed and depicted as box and whisker plots as described in Figure 2B. c-Cbl shRNA but not Rabex-5 shRNA or control shRNA decreased the numbers of LANA-positive cells.

**Figure 10:**
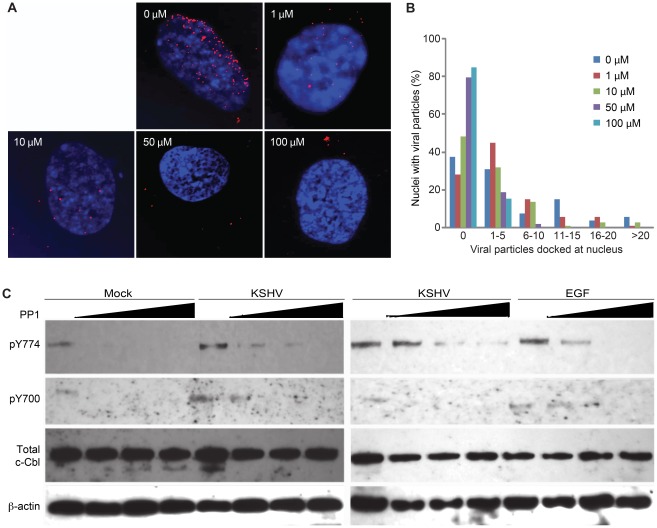
Inhibition of phosphorylation of E3 ligases reduces KSHV entry and intracellular trafficking in endothelial cells. (A) Treatment with PP1 analog inhibits KSHV entry and intracellular trafficking in a dose-dependent manner. HUVEC pretreated with PP1 analog for 1 hr, infected with KSHV for 4 hr, and stained for KSHV particles (red) and nuclei (blue). (B) Quantification of the total number of Orf65+ particles docked at each nucleus following treatment with PP1 analog. (C) Treatment with PP1 analog prevents phosphorylation of c-Cbl during KSHV infection. HUVEC pretreated with Src kinase inhibitor PP1 analog for 1 hr were infected with KSHV for 30 min. E3 ligase c-Cbl, phospho-Y700 c-Cbl, and phospho-Y774 c-Cbl were detected using β-actin as a loading control. EGF was used as a positive control for c-Cbl activation.

To directly examine the role of c-Cbl in KSHV entry and intracellular trafficking, we performed loss-of-function analyses. Knock-down of c-Cbl significantly reduced the number of KSHV particles that successfully entered the cells and trafficked to the nuclei (Figure 9A–C). In contract to c-Cbl, knock-down of Rabex-5 had no effect on KSHV entry and intracellular trafficking (Figure 9A–C). Consistent with these results, knock-down of c-Cbl but not Rabex-5 almost completely abolished KSHV infectivity as shown by the numbers of LANA-positive cells at 48 hpi (Figure 9D and Figure S4).

c-Cbl is activated through phosphorylation by the Src family kinases, and Src activity can be blocked by chemical analogs of protein phosphatase 1 (PP1) [37], which inhibits the E3 ligase activity of c-Cbl [38]. As expected, treatment of HUVEC with increasing doses of PP1 analog inhibited viral entry and intracellular trafficking, resulting in significantly fewer KSHV particles reaching the nuclei (Figure 10A–B). Under these experimental conditions, we did not observe any cytotoxicity to the cells based on PI staining (data not shown). To verify that PP1 treatment prevents c-Cbl phosphorylation, immunoblot analysis on HUVEC lysates collected following treatment with PP1 was performed (Figure 10C). Exposure of cells to either 100 ng/ml EGF or KSHV increased c-Cbl phosphorylation at tyrosines 700 and 774; however these phosphorylation modifications were abrogated in the presence of PP1 analog.

## Discussion

Our results clearly demonstrate the essential roles for both ubiquitination and proteasome functions during KSHV entry and intracellular trafficking in endothelial cells. Although it is the first time shown for KSHV infection, the ubiquitin/proteasome system is known to regulate either entry or gene expression of several other viruses, including equine infectious anemia virus [39], influenza virus [40], [41] herpes simplex 1 [42], murine coronavirus [43], and vaccinia virus [44]. We have found that the total numbers of KSHV particles reaching nuclei were significantly reduced upon suppression of proteasome function, suggesting that virus entry and intracellular trafficking was arrested at a step prior to docking at nuclei. Consistent with these results, we found that proteasome inhibitors reduced KSHV infectivity indicating that they indeed affected the KSHV infectious pathway. Three-dimensional analysis of infected cells revealed that inhibition of proteasome activity resulted in a modest increase in viral particles retained at the plasma membrane, and a significant accumulation of viral particles in the cytoplasm. Furthermore, cells treated with proteasome inhibitors had increased numbers of viral particles retained in the EEA1+ early endosomal compartments. Cells treated with proteasome inhibitors had higher numbers of EEA1+ particles at the membrane, and higher numbers of EEA1+ particles in the cytoplasm as compared to cells treated with DMSO. In contrast, proteasome inhibition reduced the total number of KSHV particles in the LAMP1+ late endosome/lysosome compartments. Lower numbers of LAMP1+ particles were observed both in the cytoplasm and perinuclear regions. These results are in agreement with the study conducted with influenza virus, which showed that proteasome activity is required for progress from the early to late endosomes during its entry of cells [40].

While the proteasome system is required for the entry and intracellular trafficking of several viruses [39]–[44], the mechanism that mediates this process remains unclear. One of the primary functions of proteasome is degradation of proteins, an intracellular event. For viruses that enter cells via endocytosis, the entry process is initiated by the attachment of infectious virions to the cellular receptors at the surface of the cell followed by internalization of viral particles and receptors. How proteasome function affects the internalization and intracellular trafficking of virions from outside the cell to the nuclei is not immediately obvious. However, it is known that internalization of EGFR and other membrane receptors requires the ubiquitination of the cytoplasmic tail. Following ubiquitination, endocytic adaptor proteins Epsin and Eps15 will recognize the ubiquitinated region of EGFR and facilitate its movement into the clathrin-coated pits. Since KSHV enters cells primarily through clathrin-mediated endocytosis [12], [13], we hypothesized that Epsin and or Eps15 are involved in the entry process. Indeed, 3-D colocalization analysis revealed that both Epsin and Eps15 are associated with KSHV particles, suggesting that either the viral particles or their receptors are ubiquitinated. In addition, when proteasome function is inhibited, the level of free ubiquitin is greatly diminished, which may explain how virus internalization and intracellular trafficking are suppressed in this context.

Viral particles require cellular structures and processes to enter cells, and specific membrane-bound receptors to facilitate their attachment, internalization, and trafficking through the intracellular space to the final destination. KSHV has evolved to use members of the integrin family, mainly integrin α3β1 and integrin αvβ3 as its receptors [15], [16]. Integrins are heterodimers composed of α and β subunits. We observed a high percentage of colocalization of KSHV particles with integrin β1. Integrin β1 is targeted for ubiquitination following inoculation with KSHV, which is prevented by treatment with an inhibitor of the E1 activating enzyme. In addition, live-labeling of cells with antibody against integrin β1 revealed that when ubiquitination is prevented, integrin β1 is retained at the plasma membrane. Finally, entry and intracellular trafficking of KSHV was greatly reduced in endothelial cells treated with the inhibitor of E1 activating enzyme, UBEI-41, in a dose-dependent manner, proving that ubiquitination itself directly mediates viral entry and trafficking. Together these results indicate that inhibition of proteasome function causes an arrest at an early stage of KSHV entry into cells, which is supported by the observations of increased numbers of viral particles at the membrane and cytoplasm, and increased colocalization of viral particles with EEA1. These results suggest that ubiquitination may be required for the initial steps of internalization from the plasma membrane and maturation of the early endosome. In fact, ubiquitination may be required at multiple steps of the entry process, from the formation of the clathrin-coated pits and internalization, to endosomal maturation, and finally docking at the nuclear membrane. Further studies should delineate the possible roles for ubiquitination during post-early endosome stages.

We have examined E3 ligases that may mediate KSHV internalization and maturation of KSHV-containing endosomes. EEA1 is a docking/tethering protein that binds to membranes containing Rab5 GTPase, thus effecting endosomal fusion and maturation [45]–[47]. Rab5 activation effected by GDP to GTP conversion is regulated by a guanine nucleotide exchange factor (GEF), Rabex-5. Rabex-5 is autoubiquitinated [48], and is also an E3 ligase with a N-terminal ubiquitin-binding domain that mediates cargo targeting to the early endosomal membrane [31], [49]. The fact that we have observed an accumulation of viral particles in an EEA1+ early endosomal compartment in conditions when ubiquitin is unavailable, as well as colocalization of viral particles with Rabex-5 is intriguing. Although the level of colocalization was minimal (6% of KSHV particles), the infection was unsynchronized, and the assay was performed at a single time point, at 4 hpi. However, we did observe substantial colocalization with the E3 ligase c-Cbl, and more specifically, we observed colocalization with the activated phosphorylated forms of c-Cbl, phospho-Y700 and phospho-Y774. Consistent with these results, knock-down of c-Cbl significantly inhibited viral entry and intracellular trafficking, and prevented KSHV infection of the endothelial cells. Furthermore, when c-Cbl activation was prevented by inhibition of Src, KSHV entry and intracellular trafficking was dramatically reduced. Immunoblot analysis confirmed that Src inhibition did in fact prevent phosphorylation of c-Cbl at tyrosine residues 700 and 774 during KSHV infection. These results are in agreement with a recent study that demonstrated the important role of c-Cbl tyrosine phosphorylation for KSHV entry into human dermal microvascular endothelial cells via the macropinocytic pathway [50]. The same group also investigated the ubiquitination of myosin IIA during KSHV infection, and found that c-Cbl may be mediating the formation of membrane blebs through its E3 ligase activity. However, our results have demonstrated the role of the ubiquitin proteasome system during clathrin-mediated KSHV endocytic entry into endothelial cells. We have independently identified c-Cbl as a mediator of KSHV entry into endothelial cells, confirming its essential roles as both an adaptor molecule and as an E3 ligase in clathrin-mediated endocytosis. Nevertheless, it remains possible that ubiquitination and other E3 ligases are involved at later stages of endosomal maturation. Mammalian cells express hundreds of E3 ligases. The functions of most of these other ligases have not been well-studied. The identification of an E3 ligase that specifically mediates ubiquitination leading to viral entry could certainly provide a potential therapeutic target.

## Materials and Methods

### Cell Culture, Viral Stock and Viral Infection

Early passage of HUVEC were obtained from Clonetics, Lonza and maintained in complete EBM-2 culture media (Allendale, NJ). KSHV-infected BCP-1 cells established from the peripheral blood mononuclear cells of a PEL patient [51] were maintained in culture in RPMI1640 containing 10% fetal bovine serum (FBS). Doxycycline-inducible iSLK cells, provided by Dr. Jae Jung at the University of Southern California, were maintained in DMEM supplemented with 10% FBS, 250 µg/ml of G418, 1,200 µg/ml of hygromycin, 1 µg/ml of puromycin, and 1% penicillin-streptomycin solution.

To induce virus production from BCP-1 cells, cells grown to log-phase were serum-starved overnight in RPMI1640. FBS was added back to the culture media to a final concentration of 10%, together with 12-*O*-tetradecanoyl-phorbol-13-acetate (TPA) at 30 ng/ml and sodium butyrate at 200 µM. At 2 days post-induction, the cells were washed and the media was replaced with fresh RPMI1640 containing 10% FBS without TPA or sodium butyrate. The culture medium was collected at 6 days post-induction. To obtain virus from iSLK cells, cells were induced with 1 µg/ml doxycycline and 1 mM sodium butyrate in DMEM supplemented with 10% FBS and the culture medium was collected 4 days later. To concentrate virus, the culture medium was centrifuged first at 5,000× g for 30 min to eliminate cell debris and then passed through a 0.45-µm filter, followed by centrifugation at 100,000× g for 3 h. The final pellet was dissolved in the culture medium overnight and was adjusted to a desired volume. Undissolved debris was eliminated by centrifugation at 5,000× g for 10 min. All the procedures for virus concentration were carried out at 4°C. The concentrated virus preparations were aliquoted and stored at −80°C for later experiments.

For cell infection, HUVEC were seeded onto glass cover slips overnight to achieve 70–80% confluency. For assays using chemical inhibitors, cells were pretreated with the inhibitors in EBM-2 media for 1 hr prior to infection. The cells were then inoculated with the virus preparation and incubated for the indicated times in the presence of the inhibitors, fixed in 2% paraformaldehyde and processed for immunostaining of KSHV particles.

### Antibodies and Chemical Inhibitors

A monoclonal antibody isotype IgG2a (clone 6A) to KSHV small capsid protein (Orf65) was used to stain KSHV particles [52]. Rabbit antibodies to EEA1, integrin β1, epsin, and eps15 were purchased from Abcam (Cambridge, MA). A rabbit polyclonal antibody against LAMP1 was purchased from Sigma Life Science (St. Louis, MO). Rabbit monoclonal antibodies against c-Cbl, and pY700 c-Cbl were from Epitomics (Burlingame, CA). A rabbit antibody against pY774 c-Cbl was from Cell Signaling (Danvers, MA). Mouse monoclonal antibodies to Cbl and Rabex-5 from Santa Cruz Biotechnology (Santa Cruz, CA) were also used for Western-blot detection. A monoclonal antibody against ubiquitin (P4D1) was from Santa Cruz. A rat anti-LANA monoclonal antibody was purchased from Abcam (Cambridge, MA).

Secondary antibodies AlexaFluor 568 goat-anti-mouse IgG1 and IgG2a, AlexaFluor 647 goat anti-mouse IgG1, AlexaFluor 488 goat anti-rabbit, and AlexaFluor 568 goat-anti-rat were from Molecular Probes, Invitrogen (Carlsbad, CA). DAPI was from BioChemika Ultra, Sigma.

Chemical inhibitors of proteasome function MG132 and EPOX were purchased from Sigma. The inhibitor of E1 activating enzyme UBEI-41 was obtained from Biogenova (Rockville, MD). Chemical inhibitor of Src kinases pp1 was from Sigma. All chemical inhibitor stock solutions were prepared according to the manufacturer's directions.

### Transduction of shRNA Lentiviruses and Knock-Down of c-Cbl and Rabex-5

c-Cbl, Rabex-5 and control shRNA lentivirus particles were purchased from Santa Cruz. HUVEC grown to 40–50% confluency in 6-well plates were pretreated with 5 µg/ml polybrene for 30 min and infected with the specified lentivirus particles. The infected cells were centrifuged at 2,000 rpm for 1 hr to facilitate virus entry, and then incubated overnight. The virus were removed on the second day, replaced with growth medium and cultured for another 3 days before inoculation with KSHV or lysed for Western-blotting detection of protein expression.

### Microscopy

For immunofluorescence analysis, cells were incubated with each primary antibody for 1 hr and then the appropriate secondary antibody conjugated to Alexa Fluor 488, 568, or 647 (Invitrogen), all at 1∶100 dilution. After washing with PBS, cells were stained with DAPI and mounted onto glass slides with FluorSave (Invitrogen). For quantification of entry and intracellular trafficking of viral particles to the nuclei, images were acquired using a Zeiss Axiovert 200M epifluorescence microscope equipped with a 63× oil immersion objective (Carl Zeiss Microimaging Inc., Thornwood, NY). Images were acquired for at least 5 fields of view per coverslip to allow counting of Orf65+ viral particles docked at nuclei. For colocalization experiments, images were acquired with an Olympus FV1000 scanning confocal microscope equipped with a 60× NA 1.42 oil immersion objective (Olympus Life Science, Center Valley, PA). Z-stacks were acquired at 0.25 µm per slice by sequentially scanning and in 8 bit color depth. Theoretical×and y axis resolution was 0.2 µm and 0.2 µm, respectively. Olympus FV1000 software was used to generate cross-sectional images and 3D-projection images (Olympus Life Science). Fluorescence images were acquired sequentially, using a 405-nm laser line with emission at 461 nm for DAPI; a 488-nm laser line with emission at 520 nm for Alexa Fluor 488; and a 543-nm laser line with emission at 603 for Alexa Fluor 568, and a 633-nm laser line with emission at 668 for AlexaFluor 647. Voltage, gain, and offset were adjusted to prevent bleed-through. Images were assembled using Adobe Photoshop CS3 version 10 (Adobe Systems Incorporated, San Jose, CA).

### Three-Dimensional Colocalization Analysis

Z-stack images were first deconvolved with AutoQuant deconvolution software using the adaptive point spread function (Media Cybernetics, Inc., Bethesda, MD). Deconvolved images were then analyzed with Imaris 3-D image analysis software (Bitplane, Zurich, Switzerland). The threshold for each channel was automatically calculated by the program using the Pearson's coefficient approach following orthogonal regression analysis on the image's scatterplot. To measure colocalization of endosomal markers with KSHV particles through the x, y, and z planes, the red channel (Orf65) was masked to create a new channel that encapsulates the 500 nm region of the image around the center of each viral particle. The masked channel was then used to determine viral particle colocalization with each of the endosomal markers (EEA1 or LAMP1) as well as for epsin, eps15, Rabex-5, c-Cbl, Y700-c-Cbl, and Y774-c-Cbl. The same procedure was also used to determine colocalization of viral particles with integrin β1 and the plasma membrane labeled with AlexaFluor647 wheat germ agglutinin (WGA) in the far-red channel as well as viral particles that were colocalized with nuclei stained with DAPI in the blue channel. The total number of colocalized pixels (voxels in 3D) was counted for cells that had a minimum of five viral particles per cell. To avoid an overrepresentation of colocalization, only one colocalization event was counted for each viral particle.

### Immunoblotting

To detect ubiquitin, HUVEC were pretreated for 1 hr with the indicated chemical inhibitors of proteasome function then inoculated with KSHV. Cell lysates were collected at the indicated time post-infection and subjected to SDS-PAGE and immunoblotting to detect ubiquitin.

For analysis of c-Cbl phosphorylation, HUVEC were serum-starved for 4 hr to reduce basal levels of c-Cbl phosphorylation, and pretreated with increasing concentrations of PP1 analog for 1 hr prior to inoculation with KSHV. Cells treated with 100 ng/ml EGF were included as a positive control for c-Cbl phosphorylation. Lysates were collected and subjected to SDS-PAGE and followed by immunoblot detection for total c-Cbl and the indicated phosphorylated forms of c-Cbl.

### Co-Immunoprecipitation Analysis

HUVEC cells were pretreated with DMSO or 5 µm UBEL-41 for 1 hr prior to inoculation with KSHV for 1 hr. Cells were washed 3 times with cold PBS, harvested by centrifugation and suspended in lysis buffer containing 20 mM Tris pH 7.4, 100 mM NaCl, 1% NP-40, 1 mM EDTA, 1 mM EGTA and protease inhibitor cocktail (sigma). Lysates were sonicated at 25% efficiency for 3 times, each for 5 sec, and centrifuged for 10 min at 10,000 g at 4°C. The lysates were pre-cleared with protein G beads for 1 hr and incubated with mouse anti-integrin β1 for 2 hr at 4°C. The protein G beads were used to precipitate the immune complexes overnight 4°C with rotation, and the immune complexes were analyzed by SDS-PAGE and Western-blotting to detect ubiquitin.

### Statistical Analysis

Results of analysis of viral particles trafficking to nuclei in the presence of chemical inhibitors were expressed as the mean ±s.d. Data were analyzed using t-test, analysis of variance, and Mann-Whitney rank-sum tests where appropriate, with *p*<0.05 considered as significant using SigmaPlot 11.0 (Systat Software, Inc., San Jose, CA). Cells bearing a minimum of five viral particles per cell were included in the analysis. The distributions of viral particle localization are summarized in box and whisker plots to represent the median values (middle lines), the 75th and 25th percentiles (opened boxes), and the 90th and 10th percentiles (short lines). Outliers outside the 90th and 10th percentiles are represented as black dots.

## Supporting Information

Figure S1 **Proteasome inhibitors MG132 and EPOX, and E1 ligase inhibitor UBEI-41 do not affect the numbers of cell-associated KSHV particles per cell.** HUVEC treated with DMSO, MG132, EPOX or UBEI-41 were inoculated with KSHV for 4 hr, and examined for total numbers of cell-associated viral particles. Box and whisker plots depict the statistical analyses of the cellular localization of KSHV particles as described in Figure 3. *p*-values <0.05 are statistically significant.
(TIF)

Figure S2 **Minimal colocalization of integrin β1 with RRV, transferrin and cholera toxin B.** To detect RRV colocalization with integrin β1, cells infected with RRV-RFP for 4 hr were stained for RRV particles (red), integrin β1 (green) and nuclei (blue). To detect colocalization or transferrin or cholera toxin B with integrin β1, cells incubated with AlexaFluor 647-transferrin (red) or AlexaFluor 647-cholera toxin B (red) for 1 hr were stained for integrin β1 (green) and nuclei (blue). Images were subjected to colocalization analysis.
(TIF)

Figure S3 **KSHV particles are colocalized with E3 ligase c-Cbl or its activated phosphorylated forms but, to a less extent, with Rabex5.** HUVEC infected with KSHV for 4 hr were stained for KSHV particles (red), E3 ligase Rabex5 (green), c-Cbl or its phosphorylated forms (pY700 or pY774) (green), and cell nuclei (blue). Z-stack images were acquired and used for colocalization analysis.
(TIF)

Figure S4 **Knock-down of c-Cbl but not Rabex-5 prevents KSHV infection of endothelial cells.** HUVEC grown on coverslips were mock treated or infected with c-Cbl, Rabex-5 or control lentivirus particles for 4 days, and infected with KSHV. Cells were fixed and stained for LANA (red) and nuclei (blue) at 48 hpi. The results were analyzed and presented in Figure 9D. c-Cbl shRNA but not Rabex-5 shRNA or control shRNA decreased the numbers of LANA-positive cells.
(TIF)

Video S1 **An overview of a HUVEC infected by KSHV.** HUVEC treated with DMSO were infected with KSHV for 4 hr, stained for KSHV particles (red), cell membrane (white) and nuclei (blue). Z-stack images were acquired with confocal laser-scanning microscopy, and deconvolved. Imaris image analysis software was used to generate 3-D contoured images and determine the localizations of viral particles in relation to the cell membrane, the cell interior, and the cell nucleus.
(MOV)

Video S2 **An overview of a HUVEC infected by KSHV in the presence of proteasome inhibitor MG132.** HUVEC treated with MG132 were processed as described in Video S1.
(MOV)

Video S3 **An overview of a HUVEC infected by KSHV in the presence of proteasome inhibitor EPOX.** HUVEC treated with EPOX were processed as described in Video S1.
(MOV)

Video S4 **Formation of a KSHV-containing vesicle during infection of HUVEC.** A region of interest from Video S1. The Imaris clipping plane function was used to reveal KSHV particles enclosed within a membrane-bound vesicle.
(MOV)

Video S5 **Association of KSHV particles with early endosomal marker EEA1.** HUVEC treated with DMSO were infected with KSHV for 4 hr, stained for viral particles (red), EEA1 (green), and nuclei (blue). Z-stacks were deconvolved and Imaris image analysis software was used to generate 3-D contoured images.
(MOV)

Video S6 **Association of KSHV particles with early endosomal marker EEA1 in the presence of proteasome inhibitor MG132.** HUVEC treated with MG132 were processed as described in Video S5.
(MOV)

Video S7 **Association of KSHV particles with early endosomal marker EEA1 in the presence of proteasome inhibitor EPOX.** HUVEC treated with EPOX were processed as described in Video S5.
(MOV)

Video S8 **Formation of KSHV-containing EEA1+ early endosomes during infection of HUVEC.** A region of interest from Video S5. The Imaris clipping plane function was used to reveal KSHV particles enclosed within an EEA1+ compartment.
(MOV)

Video S9 **Association of KSHV particles with late endosomal marker LAMP1.** HUVEC treated with DMSO were infected with KSHV for 4 hr, stained for KSHV particles (red), LAMP1 (green), and nuclei (blue). Z-stacks were deconvolved and Imaris image analysis software was used to generate 3-D contoured images.
(MOV)

Video S10 **Association of KSHV particles with late endosomal marker LAMP1 in the presence of proteasome inhibitor MG132.** HUVEC treated with MG132 were processed as described in Video S9.
(MOV)

Video S11 **Association of KSHV particles with late endosomal marker LAMP1 in the presence of proteasome inhibitor EPOX.** HUVEC treated with EPOX were processed as described in Video S9.
(MOV)

Video S12 **Formation of KSHV-containing LAMP1+ late endosomes during infection of HUVEC.** A region of interest from Video S9. The Imaris clipping plane function was used to reveal KSHV particles enclosed within an LAMP1+ vesicle.
(MOV)
