# Supplementary figures and images for: The Ubiquitin/Proteasome System Mediates Entry and Endosomal Trafficking of Kaposi's Sarcoma-Associated Herpesvirus in Endothelial Cells
Source: PLoS Pathog. 2012 May 17;8(5):e1002703. doi: 10.1371/journal.ppat.1002703 (PMC3355089; doi:10.1371/journal.ppat.1002703)

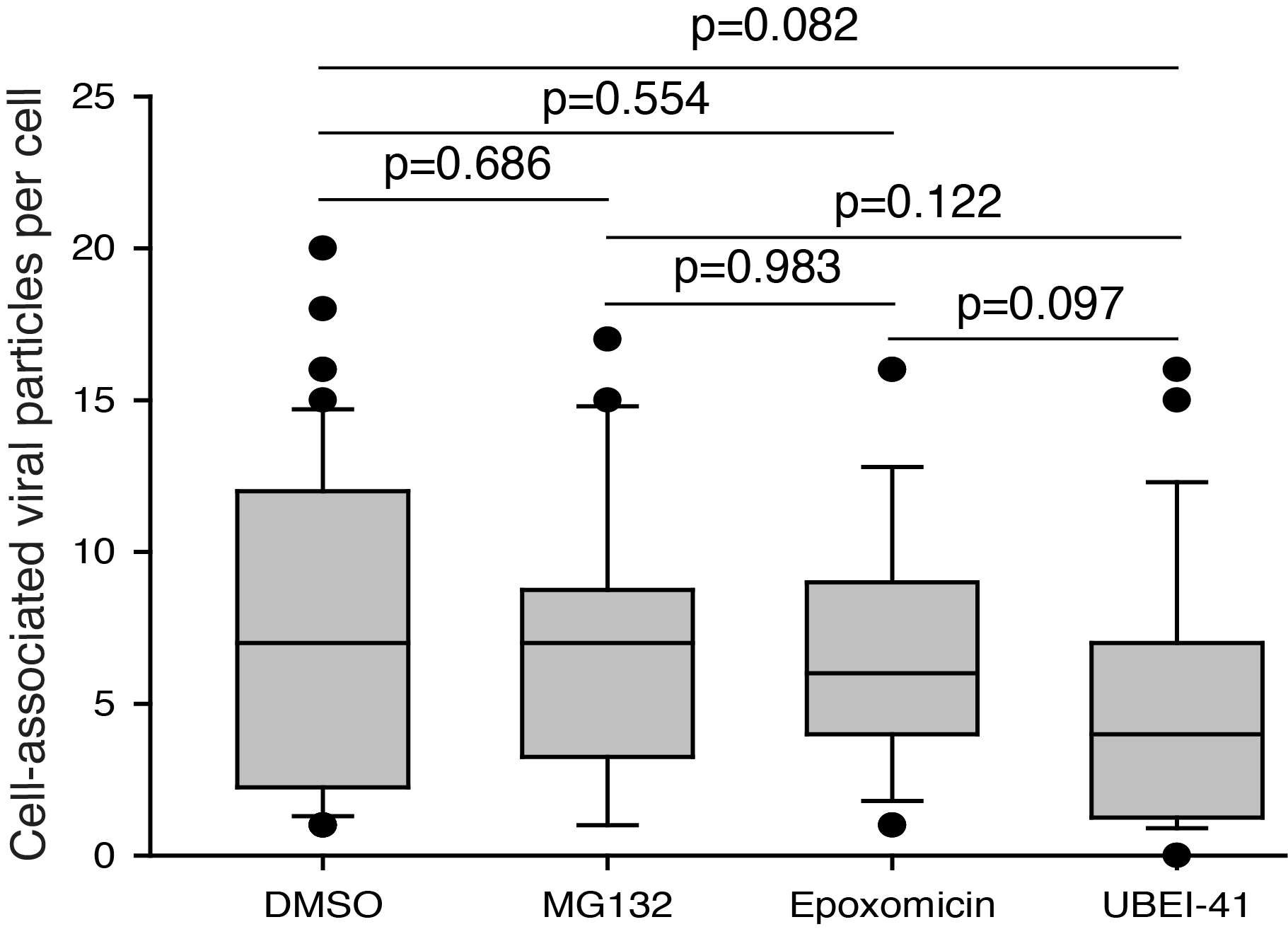

Supplement: Figure S1 — Proteasome inhibitors MG132 and EPOX, and E1 ligase inhibitor UBEI-41 do not affect the numbers of cell-associated KSHV particles per cell. HUVEC treated with DMSO, MG132, EPOX or UBEI-41 were inoculated with KSHV for 4 hr, and examined for total numbers of cell-associated viral particles. Box and whisker plots depict the statistical analyses of the cellular localization of KSHV particles as described in Figure 3. p-values <0.05 are statistically significant. (TIF) [file ppat.1002703.s001.tif]

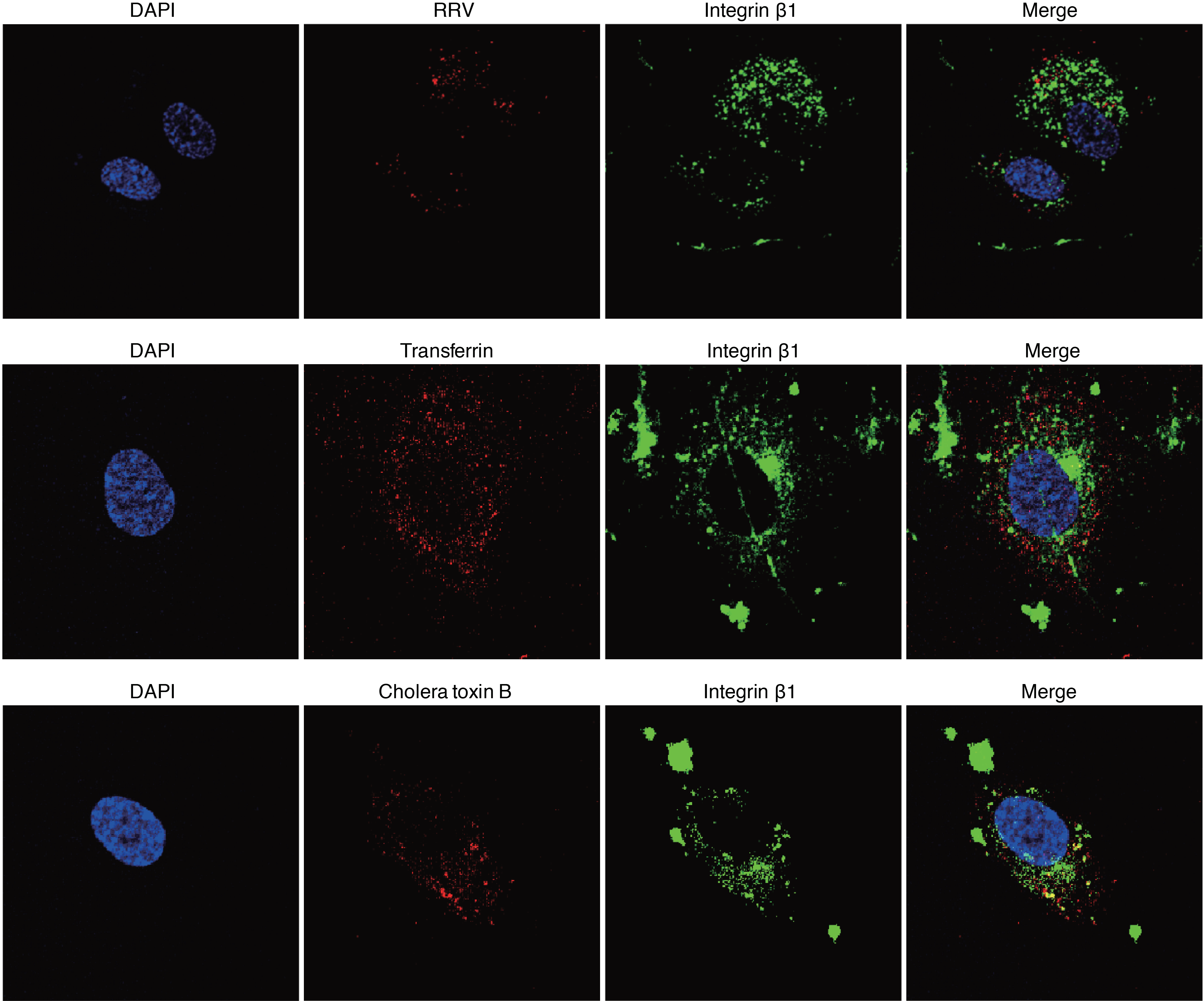

Supplement: Figure S2 — Minimal colocalization of integrin β1 with RRV, transferrin and cholera toxin B. To detect RRV colocalization with integrin β1, cells infected with RRV-RFP for 4 hr were stained for RRV particles (red), integrin β1 (green) and nuclei (blue). To detect colocalization or transferrin or cholera toxin B with integrin β1, cells incubated with AlexaFluor 647-transferrin (red) or AlexaFluor 647-cholera toxin B (red) for 1 hr were stained for integrin β1 (green) and nuclei (blue). Images were subjected to colocalization analysis. (TIF) [file ppat.1002703.s002.tif]

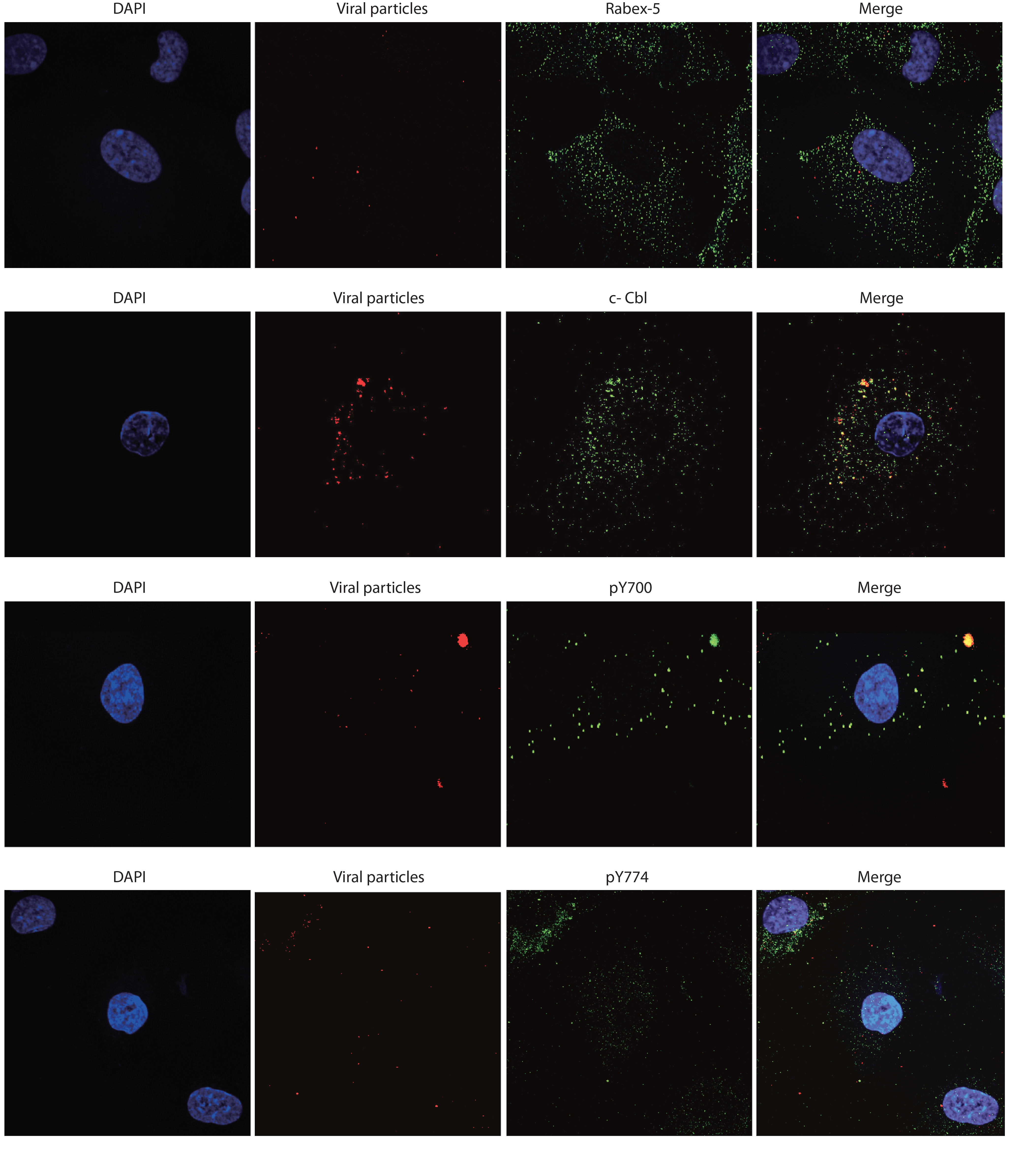

Supplement: Figure S3 — KSHV particles are colocalized with E3 ligase c-Cbl or its activated phosphorylated forms but, to a less extent, with Rabex5. HUVEC infected with KSHV for 4 hr were stained for KSHV particles (red), E3 ligase Rabex5 (green), c-Cbl or its phosphorylated forms (pY700 or pY774) (green), and cell nuclei (blue). Z-stack images were acquired and used for colocalization analysis. (TIF) [file ppat.1002703.s003.tif]

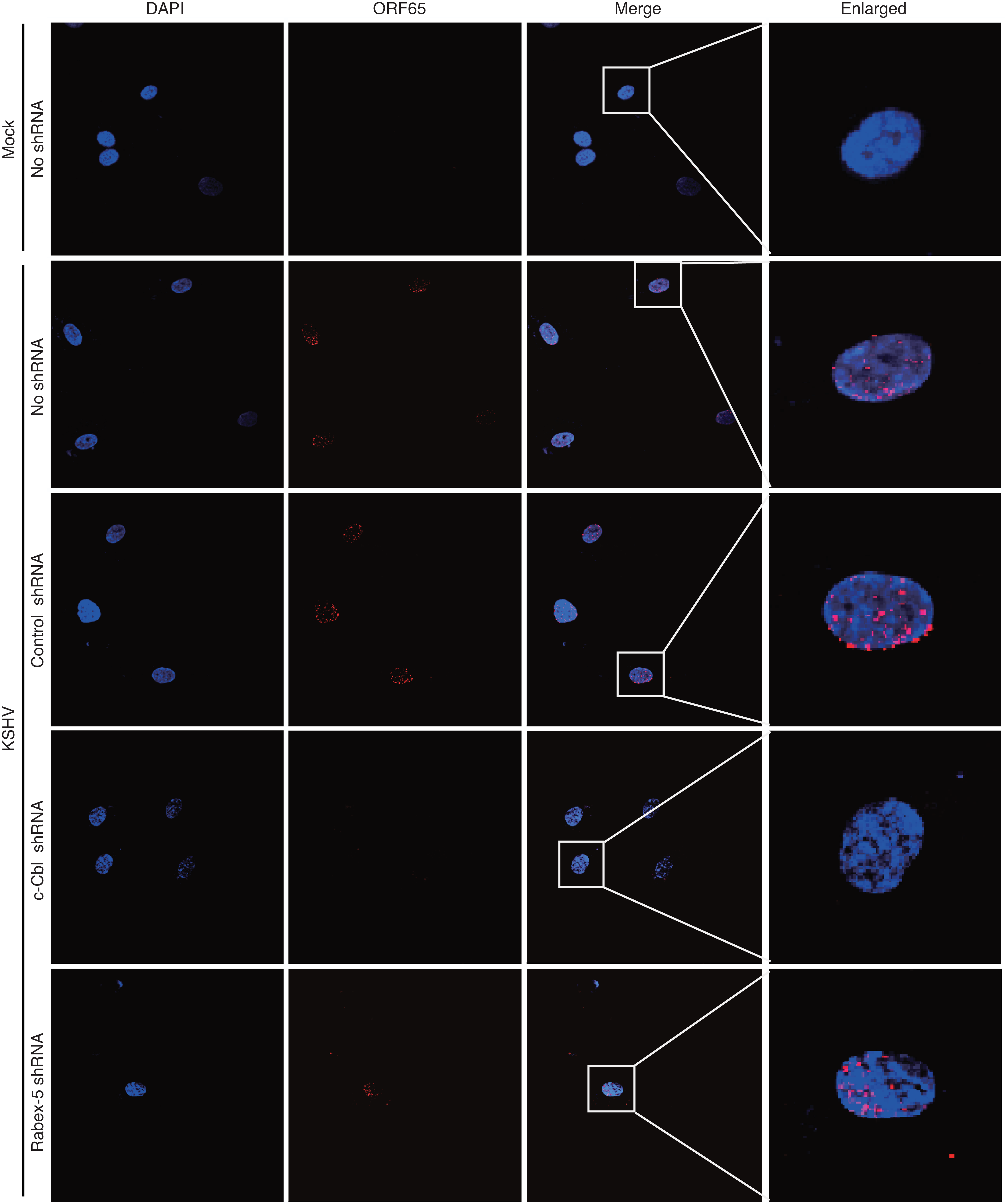

Supplement: Figure S4 — Knock-down of c-Cbl but not Rabex-5 prevents KSHV infection of endothelial cells. HUVEC grown on coverslips were mock treated or infected with c-Cbl, Rabex-5 or control lentivirus particles for 4 days, and infected with KSHV. Cells were fixed and stained for LANA (red) and nuclei (blue) at 48 hpi. The results were analyzed and presented in Figure 9D. c-Cbl shRNA but not Rabex-5 shRNA or control shRNA decreased the numbers of LANA-positive cells. (TIF) [file ppat.1002703.s004.tif]
